# Randomized Trial Comparing the Effects of Ticagrelor Versus Clopidogrel on Myocardial Perfusion in Patients With Coronary Artery Disease

**DOI:** 10.1161/JAHA.117.005894

**Published:** 2017-05-02

**Authors:** Matthieu Pelletier‐Galarneau, Chad R. R. N. Hunter, Kathryn J. Ascah, Rob S. B. Beanlands, Girish Dwivedi, Robert A. deKemp, Benjamin J. W. Chow, Terrence D. Ruddy

**Affiliations:** ^1^ Division of Nuclear Medicine The Ottawa Hospital Ottawa Canada; ^2^ Department of Medicine University of Ottawa Canada; ^3^ Division of Cardiology University of Ottawa Heart Institute Ottawa Ontario Canada

**Keywords:** adenosine, clopidogrel, myocardial blood flow, positron emission tomography, ticagrelor, Clinical Studies, Coronary Circulation, Nuclear Cardiology and PET, Coronary Artery Disease

## Abstract

**Background:**

Ticagrelor is a P2Y_12_ receptor inhibitor used in acute coronary syndromes to reduce platelet activity and to decrease thrombus formation. Ticagrelor is associated with a reduction in mortality incremental to that observed with clopidogrel, potentially related to its non–antiplatelet effects. Evidence from animal models indicates that ticagrelor potentiates adenosine‐induced myocardial blood flow (MBF) increases. We investigated MBF at rest and during adenosine‐induced hyperemia in patients with stable coronary artery disease treated with ticagrelor versus clopidogrel.

**Methods and Results:**

This randomized double‐blinded crossover study included 22 patients who received therapeutic interventions of ticagrelor 90 mg orally twice a day for 10 days and clopidogrel 75 mg orally once a day for 10 days, with a washout period of at least 10 days between the treatments. Global and regional MBF and myocardial flow reserve were measured using rubidium 82 positron emission tomography/computed tomography at baseline and during intermediate‐ and high‐dose adenosine. Global MBF was significantly greater with ticagrelor versus clopidogrel (1.28±0.55 versus 1.13±0.47 mL/min per gram, *P*=0.002) at intermediate‐dose adenosine and not different at baseline (0.65±0.19 versus 0.60±0.15 mL/min per gram, *P*=0.084) and at high‐dose adenosine (1.64±0.40 versus 1.61±0.19 mL/min per gram, *P*=0.53). In regions with impaired myocardial flow reserve (<2.5), MBF was greater with ticagrelor compared with clopidogrel during intermediate and high doses of adenosine (*P*<0.0001), whereas the differences were not significant at baseline.

**Conclusions:**

Ticagrelor potentiates global and regional adenosine‐induced MBF increases in patients with stable coronary artery disease. This effect may contribute to the incremental mortality benefit compared with clopidogrel.

**Clinical Trial Registration:**

URL: http://www.clinicaltrials.gov. Unique identifier: NCT01894789.

## Introduction

Ticagrelor is a reversible P2Y_12_ adenosine diphosphate receptor blocker. When added to aspirin for 1 year in acute coronary syndrome (ACS) patients, ticagrelor reduced major cardiovascular events compared with clopidogrel.^1^ Ticagrelor was also shown to significantly reduce the risk of major cardiovascular events including cardiovascular death, myocardial infarction, and stroke in patients with previous history of myocardial infarction.^2^, ^3^ Although superiority of ticagrelor was predictable because it is a stronger antiplatelet agent than clopidogrel,^4^, ^5^, ^6^ the observed improved survival may not have occurred given increased bleeding, as was observed in a previous study comparing clopidogrel and prasugrel.^7^ The difference in benefits between prasugrel and ticagrelor led investigators to suggest that ticagrelor may have other non–antiplatelet pleiotropic effects that contributed to the observed improve survival.

These pleiotropic properties of ticagrelor may be mediated through modulation of the adenosine plasma levels (APLs).^8^ First, ticagrelor has been shown to potentiate the effect of adenosine on myocardial blood flow (MBF). In a canine model of occlusion and hyperemia, ticagrelor potentiated reactive hyperemia and adenosine‐induced coronary flow increase.^9^ In humans, ticagrelor enhances the adenosine‐induced increase in coronary blood flow velocity in the left anterior descending coronary artery measured with transthoracic Doppler echocardiography in normal healthy volunteers compared with placebo^10^ and in patients with non–ST‐segment–elevation ACS compared with prasugrel.^11^ Second, ticagrelor increases endogenous APLs by inhibition of adenosine uptake by human erythrocytes,^9^ and higher APLs have been measured in ACS patients receiving ticagrelor compared with clopidogrel.^12^, ^13^ Because adenosine has been associated with reduction of ischemia/reperfusion injury in the peri‐infarct myocardium in patients with ACS,^14^ ticagrelor could play an important role in salvaging jeopardized tissue or reducing ischemia‐related arrhythmic events in ACS by increasing the APL.

The effects of ticagrelor on myocardial perfusion have not been studied in patients with stable coronary artery disease (CAD). The purpose of this study is to determine whether ticagrelor can increase the adenosine‐induced MBF augmentation in patients with stable CAD in comparison to clopidogrel at intermediate adenosine dosage, using positron emission tomography (PET), an accurate and reproducible quantitative tool to measure MBF.^15^, ^16^ We report the results of a phase II, single‐center, randomized, double‐blind, crossover study comparing the effects of ticagrelor versus clopidogrel on global and regional MBF and myocardial flow reserve (MFR) measured with PET in CAD patients.

## Methods

### Patient Population

Adult patients with CAD were identified, screened, and recruited from the cardiology clinics of the University of Ottawa Heart Institute. Inclusion criteria were stable CAD, age ≥18 years, and body mass index (kg/m^2^) ≤30. Exclusion criteria were contraindication to use of clopidogrel, ticagrelor, or aspirin; anticoagulation therapy; history of intracranial bleeding; recent pathological bleeding; significant arrhythmias; moderate to severe hepatic impairment; dyspnea (New York Heart Association classes III–IV); revascularization within 90 days; ACS within 60 days; any scheduled surgery during the trial period; concomitant therapy with a strong cytochrome CYP3A inhibitor or inducer; recent use of dipyridamole; known hypersensitivity to ticagrelor, clopidogrel or adenosine; breast feeding or pregnancy; and aspirin maintenance dose >100 mg by mouth daily.

Sample size estimations were based on our previous work showing a repeatability coefficient for stress MBF to be 0.46 mL/min per gram, consistent with a within‐participant standard deviation between the 2 known values of the same patient of 0.23 mL/min per gram.^15^ To allow a minimal detectable difference of stress MBF between treatments of 5% (0.15 mL/min per gram assuming a stress MBF of 3 mL/min per gram) in a 2‐treatment crossover study with power of 0.80 and α of 0.05, the study required 22 participants; therefore, 25 participants were included to allow for 3 incomplete studies. The study was conducted in accordance with the International Conference on Harmonization Guidelines and the Declaration of Helsinki. Participants provided written informed consent. The study protocol was approved by the Ottawa Health Science Network research ethics board.

### Study Drug Administration

**Figure 1:**
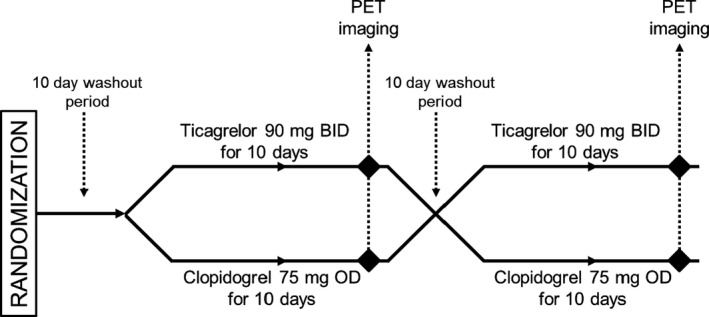
Schematic representation of randomized crossover design. BID indicates twice a day; OD, once daily; PET, positron emission tomography.

Participants were enrolled in a double‐blinded randomized crossover study during which they were randomly assigned to received 1 of 2 treatments (ticagrelor 90 mg orally twice a day for 10 days versus clopidogrel 75 mg orally daily for 10 days) and then crossed over to the other treatment (Figure 1). Medications were formulated as identical blinded capsules, and a placebo was given as the second daily dose for the clopidogrel treatment. Prior to the start of each treatment, the patients underwent a washout period of at least 10 days during which they received neither ticagrelor nor clopidogrel. PET/CT (PET and computed tomography) imaging was performed after each treatment on the 10th day of treatment. Weekly phone calls or emails were made to ensure compliance with the dosing regimen. Aspirin 81 mg daily was maintained throughout the trial.

### MBF Measurements

Prior to PET/CT imaging, vasoactive medications were held for 5 plasma half‐lives, and participants were instructed to refrain from caffeine for 24 hours. At each imaging session, 3 myocardial perfusion imaging PET scans were performed: (1) baseline acquisition, started 120 to 150 minutes after ingestion of study medication; (2) a scan with an intermediate adenosine dose of 80 μg/kg per minute for 6 minutes; and (3) a scan with a high adenosine dose of 140 μg/kg per minute for 6 minutes. There was a resting period of at least 10 minutes between the adenosine studies.

Rubidium 82 PET/CT imaging was performed using a 3‐dimensional PET/CT system (Discovery 690; GE Healthcare) following a previously described protocol.^15^ Briefly, the baseline PET scan included a low‐dose CT for attenuation correction (fast helical 1.5 seconds, 120 kVp with axial and angular mA modulation at a noise index of 50). Participants received 10 MBq/kg of Rb‐82 intravenously over 30 seconds, and PET imaging was performed over a period of 6 minutes. MBF was quantified using a 1‐tissue‐compartment model with a flow‐dependent extraction correction,^15^ and MBF polar maps were generated using a validated software (FlowQuant).^16^ MFR is calculated as adenosine stress MBF divided by baseline MBF. Left ventricular ejection fraction (LVEF) data were extracted from the gated PET images using automated 4DM‐PET software (INVIA). Regional wall motion was graded by 1 observer on a 6‐point scale (4=normal to −1=dyskinetic). Rate pressure product calculation was performed using the average hazard ratio and systolic blood pressure at peak stress and after tracer infusion. MFR corrected for rate pressure product was calculated by multiplying MFR by resting rate pressure product and dividing by 8500.^17^

### Statistical Analysis

Two‐way repeated‐measures ANOVA followed by Holm‐Sidak multiple comparisons testing was conducted to examine the effect of treatment and adenosine level on MBF, MFR, LVEF, and wall motion. For regional analyses, segments were defined using the standard 17‐segment model,^18^ and statistical analyses were conducted with 3‐way repeated‐measures ANOVA. Results are presented as mean±SD. Values of *P*<0.05 were considered statistically significant. Averages were compared using paired *t* test testing. For this crossover trial, tests for period effects and treatment–period interaction were performed using the MFR of intermediate‐ and high‐dose adenosine.^19^ Analyses were performed using GraphPad Prism version 7.00 for Windows (GraphPad Software) and IBM SPSS Statistics for Windows, version 22.0 (IBM Corp)

## Results

### Patient Characteristics

Of 1305 consecutive patients assessed for eligibility, 729 patients did not meet inclusion criteria (Table 1). Of the remaining 576 patients, 547 patients met the exclusion criteria. Consent was obtained from 29 patients, 6 withdrew from the study before participation, and 23 patients were randomized and imaged. One patient was excluded because of camera failure during image acquisition. The characteristics of the final 22 participants are shown in Table 2. No participant was on maintenance therapy with clopidogrel or ticagrelor. There were no adverse events related to the treatments during the study. There was no significant period effect (*P*=0.72) or period–treatment interaction (*P*=0.17) in this crossover study.

**Table 1:** Inclusion and Exclusion Criteria Table

Reasons for Screening Failures	
Patients screened from cardiology clinics	1305
Patients not meeting inclusion criteria	
1. Age ≥18 y	0
2. Stable coronary artery disease on stable medical treatment	488
3. Body mass index ≤30	58
4. No clinically significant abnormalities on baseline laboratory work	43
5. No clinically significant arrhythmias on baseline 12‐lead electrocardiogram	140
6. Female participants must be postmenopausal or surgically sterilized or have a negative urine β‐human chorionic gonadotropin pregnancy test at initial screening and maintain effective contraceptive methods throughout the trial and for 30 d following the end of dosing treatment	0
Total	729
Patients meeting exclusion criteria	
1. Any contraindication against the use of clopidogrel, ticagrelor, and/or reduced dose of acetylsalicylic acid	27
2. Oral anticoagulation therapy	318
3. History of intracranial bleeding	7
4. Recent or active pathological bleeding, such as peptic ulcer	32
5. Moderate or severe hepatic impairment	4
6. History or risk of bradycardia	7
7. Known second‐ or third‐degree atrioventricular block without pacemaker	1
8. Dyspnea (New York Heart Association classes III/IV), wheezing asthma, or chronic obstructive pulmonary disease	55
9. Coronary artery bypass grafting surgery within 90 d before screening or at any time after consent	1
10. Percutaneous coronary intervention within 90 d before screening or at any time following consent	1
11. Acute myocardial infarction or acute coronary syndrome within 60 d before to screening or at any time following consent	0
12. Any scheduled surgery during the trial period, including dental	4
13. Concomitant therapy with strong cytochrome CYP3A inhibitor or inducer	26
14. Recent use of dipyridamole or dipyridamole‐containing medications (eg, Aggrenox)	0
15. Known hypersensitivity to the investigational drug or any of its components	2
16. Known hypersensitivity to adenosine	0
17. Lactose intolerance	0
18. Known human immunodeficiency virus or hepatitis B or C positive	0
19. Breastfeeding or pregnancy	0
20. Claustrophobia or inability to lie still in a supine position	2
21. Unwillingness to provide informed consent	60
Total	547

**Table 2:** Participant Characteristics

Characteristic (n=22)	
Age, y	61.7±8.8
Male sex	3 (13.6%)
Height, cm	174.4±8.1
Weight, kg	81.6±9.8
Body mass index, kg/m^2^	26.9±3.5
Cardiovascular risk factors	
Smoker	
Current	3 (13.6%)
Past	11 (50.0%)
Diabetes mellitus	
Insulin dependent	2 (8.7%)
Noninsulin dependent	3 (13.6%)
Hypertension	17 (77.3%)
Hyperlipidemia	20 (90.9%)
Coronary artery disease history	
Prior hospitalization	2 (9.1%)
Previous myocardial infarct	13 (59.1%)
Previous angiogram	16 (72.7%)
Previous percutaneous coronary intervention	10 (45.5%)
Previous coronary artery bypass grafting	5 (22.7%)
Other medical history	
Chronic renal disease	0 (0%)
Peripheral vascular disease	1 (4.5%)
Average time between imaging, d	19.8±3.8 (range 16–31)

### Hemodynamics

At baseline, heart rate (62.8±10.1 versus 65.3±13.1 bpm, *P*=0.19), systolic blood pressure (128.8±22.7 versus 124.7±17.9 mm Hg, *P*=0.25), diastolic blood pressure (72.1±10.7 versus 70.9±10.1 mm Hg, *P*=0.60), and rate‐pressure product (8173±2357 versus 8235±2406, *P*=0.89) were not significantly different between the ticagrelor and clopidogrel phases.

### MBF and MFR

**Figure 2:**
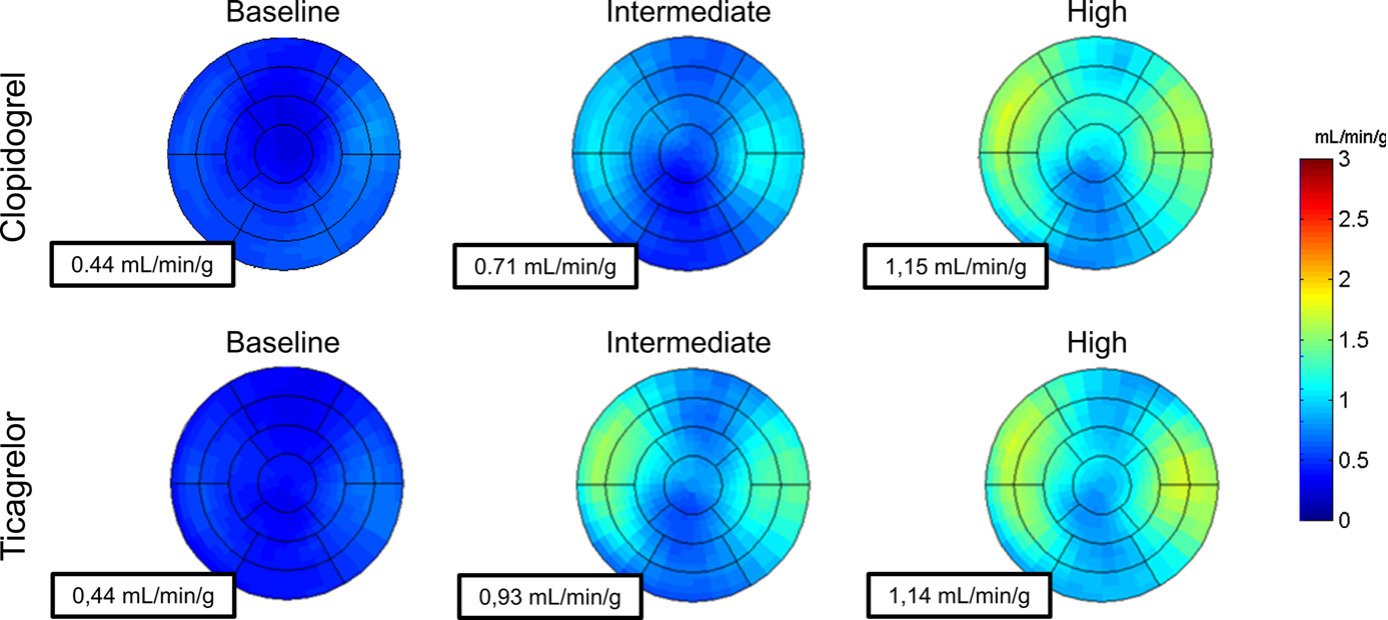
Myocardial blood flow (MBF) polar maps of a representative patient with global MBF presented for clopidogrel and ticagrelor at baseline and during intermediate and high adenosine doses. Global MBF was not different at baseline and high‐dose adenosine, whereas it was greater at intermediate adenosine dose with ticagrelor compared with clopidogrel.

**Figure 3:**
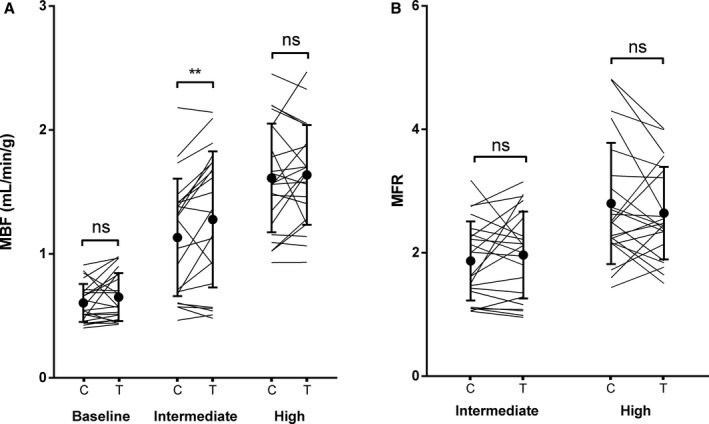
Global (A) myocardial blood flow (MBF) and (B) myocardial flow reserve (MFR) at baseline and during intermediate and high adenosine. *^*^ *P*<0.01. ns indicates not significant.

A 2‐factor repeated‐measures ANOVA revealed a significant main effect of adenosine dose on MBF (F[2, 42]=68.35, *P*<0.0001). MBF was greater with a higher dose of adenosine. The effect of medication was also significant (F[1, 21]=5.479, *P*=0. 029). MBF was greater with ticagrelor compared with clopidogrel. There was no statistically significant interaction between adenosine stress dose and treatment on MBF (F[2, 42]=2.626, *P*=0.084). MBF was significantly greater with ticagrelor compared with clopidogrel at intermediate adenosine dose (*P*=0.0020), whereas it was not different at baseline (*P*=0.43) and high‐dose adenosine (*P*=0.53). Baseline MBF corrected for rate pressure product remained not significantly different between ticagrelor and clopidogrel (0.65 versus 0.62 mL/min per gram, *P*=0.82). Average differences between ticagrelor and clopidogrel MBF were 0.05±0.15, 0.15±0.23, and 0.03±0.25 mL/min per gram at baseline, intermediate‐, and high‐dose adenosine, respectively (Figures 2 and 3, Table 3).

**Table 3:** Global and Regional MBF and MFR

	MBF (mL/min per gram)			MFR		
	Clopidogrel	Ticagrelor	*P* Value	Clopidogrel	Ticagrelor	*P* Value
Global						
Baseline	0.60±0.15	0.65±0.19	0.4254			
Intermediate	1.13±0.47	1.28±0.55	0.0020	1.83±0.65	1.94±0.70	0.2740
High	1.61±0.44	1.64±0.40	0.5302	2.80±0.97	2.74±0.87	0.1581
Regions						
MFR_h_ <1.5						
Baseline	0.64±0.19	0.65±0.15				
Intermediate	0.90±0.38	1.02±0.42		1.36±0.27	1.55±0.44	
High	0.87±0.33	1.05±0.41		1.32±0.19	1.63±0.47	
MFR_h_ 1.5 to <2.0						
Baseline	0.68±0.19	0.72±0.24				
Intermediate	1.09±0.43	1.31±0.58		1.57±0.42	1.79±0.64	
High	1.21±0.30	1.41±0.40		1.80±0.16	2.05±0.54	
2.0≤ MFR_h_ <2.5						
Baseline	0.71±0.17	0.73±0.21				
Intermediate	1.36±0.55	1.47±0.58		1.88±0.51	1.98±0.55	
High	1.59±0.38	1.71±0.48		2.26±0.13	2.42±0.54	
2.5 to <3.0						
Baseline	0.66±0.14	0.74±0.22				
Intermediate	1.23±0.54	1.41±0.66		1.84±0.67	1.96±0.85	
High	1.81±0.39	1.84±0.42		2.74±0.14	2.60±0.60	
3.0 to <3.5						
Baseline	0.62±0.19	0.74±0.30				
Intermediate	1.36±0.53	1.64±0.65		2.21±0.62	2.33±0.70	
High	2.01±0.60	2.01±0.59		3.23±0.16	2.85±0.50	
≥3.5 MFR_h_						
Baseline	0.47±0.08	0.51±0.13				
Intermediate	1.01±0.46	1.08±0.53		2.13±0.83	2.07±0.82	
High	2.01±0.39	1.79±0.46		4.29±0.55	3.50±0.61	

MBF indicates myocardial blood flow; MFR, myocardial flow reserve; MFR_h_ indicates myocardial flow reserve during high‐dose adenosine stress.

A 2‐factor repeated‐measures ANOVA revealed a significant main effect of adenosine dose on MFR (F[1, 21]=19.18, *P*=0.0003). MFR was greater with high compared with intermediate adenosine dose. The effect of medication was not significant (F[1, 21]=0.07414, *P*=0.79). MFR was not significantly different with ticagrelor compared with clopidogrel at intermediate (*P*=0.27) and high adenosine dose (*P*=0.16). However, there was a statistically significant interaction between adenosine dose and treatment on MFR (F[1, 21]=4.343, *P*=0.0496).

### Regional MBF and MFR

**Figure 4:**
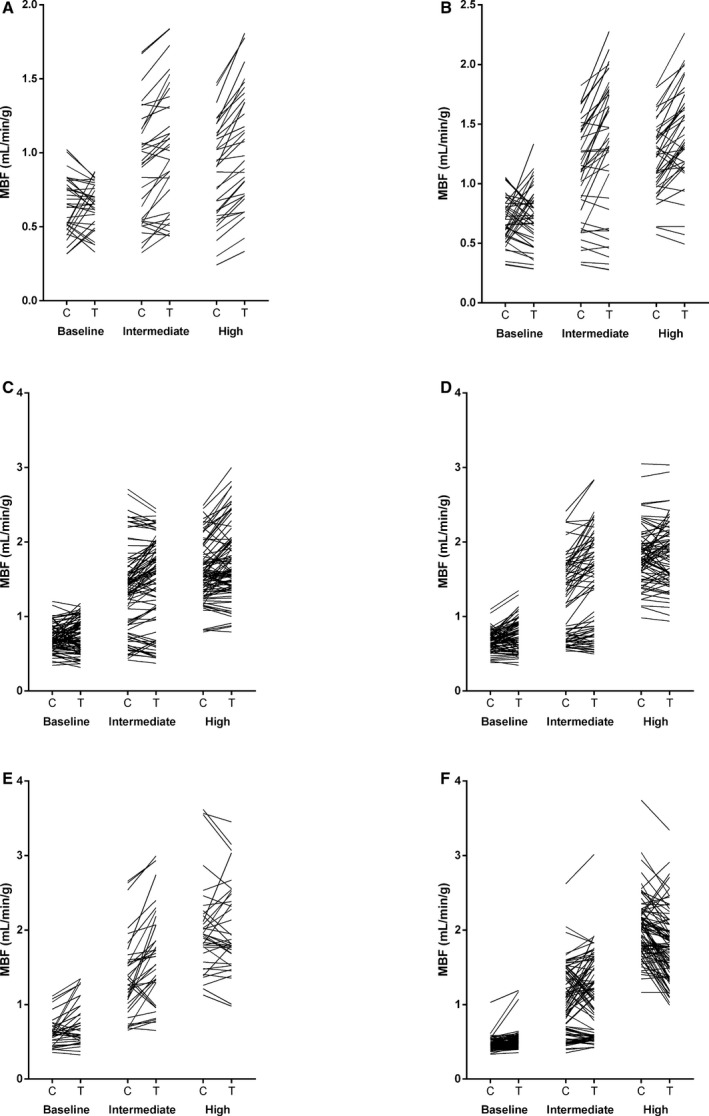
Regional myocardial blood flow (MBF) at baseline and during intermediate and high adenosine of regions with myocardial flow reserve (MFR) <1.5 (A), ≥1.5 and <2.0 (B), ≥2.0 and <2.5 (C), ≥2.5 and <3.0 (D), ≥3.0 and <3.5 (E), and ≥3.5 (F).

**Figure 5:**
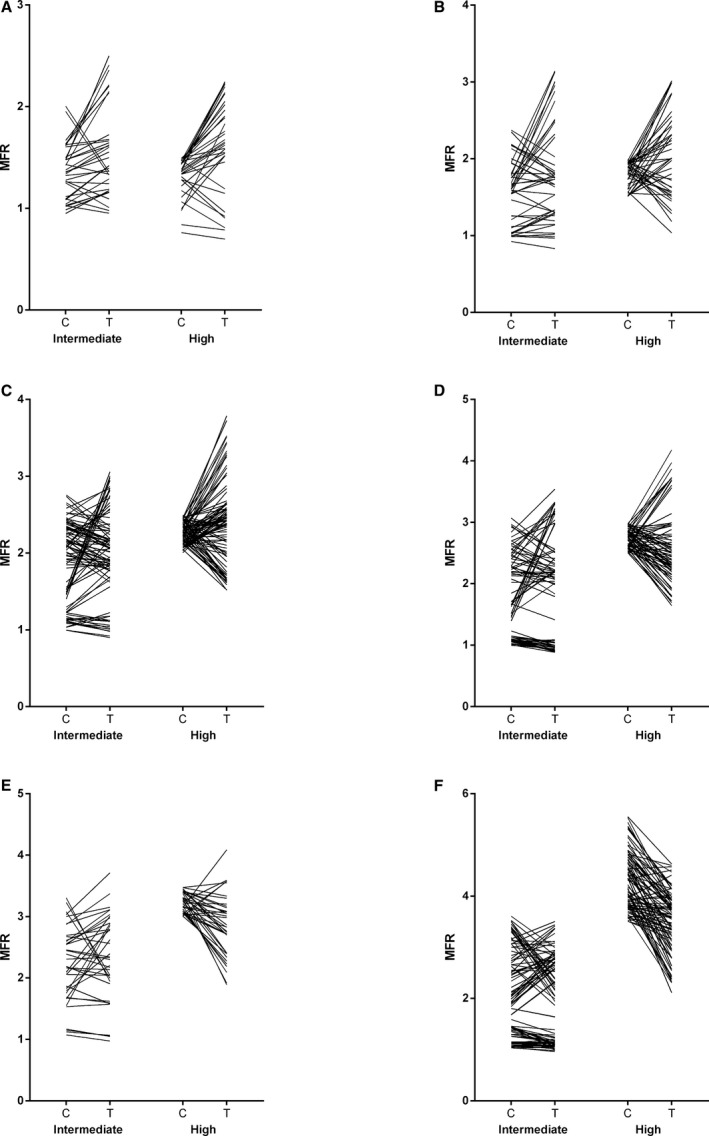
Regional myocardial flow reserve (MFR) during intermediate and high adenosine of regions with MFR <1.5 (A), ≥1.5 and <2.0 (B), ≥2.0 and <2.5 (C), ≥2.5 and <3.0 (D), ≥3.0 and <3.5 (E), and ≥3.5 (F).

For regional analyses, left ventricular regions were grouped based on MFR with high‐dose adenosine during clopidogrel treatment (MFR_h_). A total of 5, 10, 13, 13, 10, and 8 participants presented at least 1 region with MFR_h_ <1.5, ≥1.5 and <2.0, ≥2.0 and <2.5, ≥2.5 and <3.0, ≥3.0 and <3.5, and ≥3.5, respectively. A total of 34, 43, 96, 74, 37, and 90 regions had MFR_h_ <1.5, ≥1.5 and <2.0, ≥2.0 and <2.5, ≥2.5 and <3.0, ≥3.0 and <3.5, and ≥3.5, respectively (Table 3, Figures 4 and 5).

For regions with MFR_h_ <2.5, regional MBF values were greater with ticagrelor compared with clopidogrel at intermediate and high adenosine doses but not at baseline. For regions with MFR_h_ ≥2.5 and <3.5, regional MBF values were greater with ticagrelor compared with clopidogrel at baseline and during intermediate adenosine but not during high‐dose adenosine. For regions with MFR_h_ ≥3.5, regional MBFs were greater with ticagrelor compared with clopidogrel at baseline and intermediate adenosine but was greater with clopidogrel compared with ticagrelor at high adenosine dose. For regions of MFR_h_ <3.0, regional MFR values were greater with ticagrelor compared with clopidogrel at intermediate and high adenosine doses. For regions with MFR_h_ ≥3.0, regional MFR values were greater with clopidogrel compared with ticagrelor at high dose adenosine.

### Left Ventricular Function

There was no significant interaction between treatment and adenosine dose on LVEF (F[2, 42]=1.956, *P*=0.15). Simple main effects analysis showed that LVEF was greater with higher dose of adenosine (*P*<0.0001), whereas there was no significant effect of treatment on LVEF (*P*=0.082). LVEF was greater at high compared with intermediate adenosine (58.2±8.5% versus 55.6±10.7%, *P*=0.046), at high adenosine compared with baseline (52.0±8.5%, *P*<0.0001), and at intermediate adenosine compared with baseline (*P*=0.0084).

There was no significant interaction between treatment and adenosine dose on wall motion (*P*=0.83). Simple main effects analysis showed that wall motion was decreased with higher dose of adenosine (baseline 3.78±0.6, intermediate adenosine 3.77±0.60, high adenosine 3.66±0.89; *P*=0.002), whereas there was no significant effect of treatment on wall motion (*P*=0.61).

## Discussion

This study is the first to demonstrate that ticagrelor augments the adenosine‐mediated MBF increase in patients with stable CAD compared with clopidogrel and extends previous observations of enhanced coronary artery velocity with intermediate‐dose adenosine with ticagrelor in healthy participants compared with placebo^10^ and in patients with non–ST‐segment–elevation ACS compared with prasugrel.^11^ At high adenosine dosage, MBF was not significantly different between ticagrelor and clopidogrel. The fact that MBF was greater with ticagrelor at intermediate and not high adenosine dosage supports the hypothesis that ticagrelor has additional adenosine‐mediated effects compared with clopidogrel in patients with CAD. Indeed, at a dosage of 140 μg/kg per minute, the maximal effect of exogenous adenosine on MBF is reached in most patients, and further increases in adenosine concentration do not induce additional MBF rise.^20^ At high adenosine dosage, maximal adenosine effect was reached with both medications; therefore, no difference was observed. Conversely, at intermediate adenosine dosage, additional adenosine‐mediated effects of ticagrelor resulted in a supplemental increase in MBF compared with clopidogrel. The differences in MBF with ticagrelor and clopidogrel at intermediate dosage of adenosine did not result in significantly different MFR. This may be related to the fact that resting MBF was on average 8% greater with ticagrelor compared with clopidogrel, and higher rest flow yields lower MFR.

If endogenous levels of adenosine are higher with ticagrelor compared with clopidogrel, one might expect that baseline MBF would be higher at rest with ticagrelor. Although baseline MBF was 8% higher with ticagrelor versus clopidogrel, the difference was not statistically significant. This could be related to the relatively small sample size of this study and the heterogeneous population or an underlying physiological process such as downregulation of A2A adenosine receptors. For regions with reduced MFR_h_ (<2.5), ticagrelor increased regional MBF during intermediate and high adenosine doses compared with clopidogrel. For regions with MFR_h_ <3.0, ticagrelor increased regional MFR during intermediate and high adenosine doses compared with clopidogrel. These results suggest that the incremental effects of ticagrelor versus clopidogrel are more important in regions of low flow reserve. For regions of MFR_h_ >3.0, regional MBF reserve values were greater with clopidogrel compared with ticagrelor at high‐dose adenosine. This result needs to be interpreted with caution. A selection bias exists when comparing the MFR_h_ obtained with clopidogrel versus ticagrelor for regions of very high MFR_h_ only. At very high value of MBF, error in MBF measurements with rubidium is higher.^21^ This subgroup of regions is thus more prone to measurement errors and may contain outliers, rendering the analyses less reliable.

Adenosine has several beneficial biological effects on the cardiovascular system.^22^ Adenosine reduces inflammation, inhibits platelet aggregation, and has negative chronotropic effects.^23^, ^24^ Adenosine is released by the myocardium in the setting of oxygen supply–demand mismatch.^25^ During ischemia, adenosine can increase oxygen supply and decrease myocardial oxygen consumption.^25^, ^26^ Adenosine can also increase glycolytic flux, enhancing efficient energy production.^25^ In addition, chronic exposure to adenosine induces angiogenesis. These effects of endogenous adenosine, mediated by A1 and A3 receptors, may provide cardioprotection during brief and prolonged episodes of ischemia.^27^ Consequently, ticagrelor may have an incremental cardioprotective role compared with clopidogrel or prasugrel in patients with stable angina, via increased availability of endogenous adenosine. Nonetheless, adenosine can promote adverse effects on circulation, including decreased blood flow to collateral‐dependent myocardium, or coronary steal.^28^ However, the improvement in outcome observed in trials comparing ticagrelor with other antiplatelets agents without an increase in APL suggests that the beneficial effects of adenosine outweigh these possible adverse effects.

### Limitations

This study measured MBF after 10 days of treatment with ticagrelor and clopidogrel, and effects with longer duration therapy may differ. Because we did not measure adenosine levels, our data support but do not prove the concept of increased availability of adenosine during ticagrelor therapy. The sample size was small, and results need to be confirmed in a larger population. The patient group represented the usual cardiology clinic patients with chronic CAD but was heterogeneous with previous percutaneous coronary intervention in 45%, previous coronary artery bypass grafting in 22%, and previous myocardial infarction in 59%. A larger sample size would permit further subgroup analysis and possible subgroup differences. Another limitation of this study pertains to the regional analyses. By classifying the regions according to their MFR at high‐dose adenosine with the clopidogrel treatment, a bias is introduced that can potentially cause a regression to the mean effect, especially in regions of very high or very low MFR.

## Conclusion

In patients with stable CAD, treatment with clinical doses of ticagrelor augmented the increase in global MBF induced with an intermediate dose of adenosine compared with clopidogrel. Importantly, these beneficial effects with ticagrelor were present in regions with impaired MFR and with both intermediate and high doses of adenosine.

## Sources of Funding

This study was supported by a research grant from AstraZeneca Canada Inc.

## Disclosures

Beanlands is a Career Investigator supported by the Heart and Stroke Foundation of Canada, the University of Ottawa Heart Institute Vered Chair in Cardiology, and Tier 1 Chair in Cardiovascular Research from the University of Ottawa. Beanlands is or has been a consultant for and receives grant funding from GE Healthcare, Lantheus Medical Imaging and Jubilant DraxImage. deKemp is a consultant and has received grant funding from Jubilant DraxImage. deKemp receives revenues from Rubidium‐82 generator technology licensed to Jubilant DraxImage, and from sales of FlowQuant software. Chow has salary support from the University of Ottawa Heart Institute Goldfarb Chair in Cardiac Imaging. Ruddy has collaborated with and received research funding from GE Healthcare and Advanced Accelerator Applications. The other authors have no conflicts to report. Chow receives research support from CV Diagnostix and educational support from TeraRecon Inc Dwivedi is supported by a CIHR new investigator salary support award.
